# Sol-Gel Derived Active Material for Yb Thin-Disk Lasers

**DOI:** 10.3390/ma10091020

**Published:** 2017-09-02

**Authors:** Rui M. Almeida, Tiago Ribeiro, Luís F. Santos

**Affiliations:** Centro de Química Estrutural/DEQ, Instituto Superior Técnico, Universidade de Lisboa, Av. Rovisco Pais, 1049-001 Lisboa, Portugal; tiago.velez.ribeiro@tecnico.ulisboa.pt

**Keywords:** sol-gel, thin-disk laser, Yb-doped glasses, aluminosilicate glasses, photoluminescence

## Abstract

A ytterbium doped active material for thin-disk laser was developed based on aluminosilicate and phosphosilicate glass matrices containing up to 30 mol% YbO_1.5_. Thick films and bulk samples were prepared by sol-gel processing. The structural nature of the base material was assessed by X-ray diffraction and Raman spectroscopy and the film morphology was evidenced by scanning electron microscopy. The photoluminescence (PL) properties of different compositions, including emission spectra and lifetimes, were also studied. Er^3+^ was used as an internal reference to compare the intensities of the Yb^3+^ PL peaks at ~ 1020 nm. The Yb^3+^ PL lifetimes were found to vary between 1.0 and 0.5 ms when the Yb concentration increased from 3 to 30 mol%. Based on a figure of merit, the best active material selected was the aluminosilicate glass composition 71 SiO_2_-14 AlO_1.5_-15 YbO_1.5_ (in mol%). An active disk, ~ 36 μm thick, consisting of a Bragg mirror, an aluminosilicate layer doped with 15 mol% Yb and an anti-reflective coating, was fabricated.

## 1. Introduction

The use of laser technology in industrial and scientific applications is widely spread, given its added value in tasks such as welding, cutting or marking, where the speed and quality of the laser beam are determinant, thus influencing the competitiveness of the industry. Among the various types of lasers currently available, dielectric solid state lasers play an important role due mainly to the beam quality. They normally use a crystalline material as the lasing medium and, contrarily to semiconductor diode lasers, they are usually optically pumped. Fiber and disk lasers, in particular Yb^3+^-doped ones, such as Yb:YAG, present high average output power, excellent beam quality and high efficiency [1,2]. Due to their unique features, thin-disk lasers are one of the best laser solutions for material micro-processing in automotive, aerospace, and heavy industry, with laser powers of up to 16 kW and beam qualities ≥2 mm·mrad [3], or for military applications as recently demonstrated by Boeing, where more than 30 kW power was attained [4].

The thin-disk laser concept was developed by Adolf Giesen et al. [5], who used a thin laser crystal disc with one face mounted on a water cooled block. The use of a very small thickness of active material (~100–200 µm), corresponding to a high surface area to volume ratio, leads not only to superior beam quality, but also to highly efficient cooling, resulting in almost negligible thermal gradients. Moreover, the residual gradient is symmetrical, which also contributes to an optimal beam quality. However, the small thickness of the active material results in an insufficient absorption length that must be compensated by a multi-pass pumping scheme [6]. The gain medium is typically a thin disk of Yb:YAG crystal grown by the Czochralski method, or another ytterbium-doped medium such as Yb:Lu_3_Al_5_O_12_ or Yb:Lu_2_O_3_ [7,8], with a highly reflective coating on one side and an anti-reflective coating on the other side. The advantages of Yb-doped active material include the simple electronic structure of Yb^3+^, with only one excited state level (^2^F_5/2_) with a lifetime of the order of 1–2 ms and ~10,000 cm^−1^ above the ground state (^2^F_7/2_), therefore with low non-radiative decay rates in most solid matrices and the possibility of achieving high doping levels without excessive luminescence quenching [9]. However, cutting such a small crystal thickness presents problems that could be overcome with a different approach: the active medium could be prepared by sol-gel (SG) processing. This is a relatively cheap wet chemistry technique which involves the hydrolysis and condensation of precursor species like organometallic compounds and nitrates in the presence of a catalyst, forming a porous gel structure that can be densified by subsequent heat treatment. Thick films can be doped with different concentrations of an active species like Yb and deposited on an appropriate substrate, e.g., a single crystal Si wafer. Moreover, anti-reflective and highly reflective coatings can be obtained as 1-D Photonic Bandgap Structures (PBGs), or photonic crystals (PCs), which are multilayered structures at the optical nanoscale that control the propagation of light, including the inhibition or enhancement of spontaneous emission (SE) of light at certain wavelengths [10,11]. Such structures may be deposited with high optical quality by the SG process [12].

Recently, our research group obtained polycrystalline Yb:YAG ceramic films as well as Yb-doped silicate glass films by the sol-gel process, with potential application as the active material in thin-disk lasers [13,14]. In the present work, multilayered structures based on Yb-doped SiO_2_-Al_2_O_3_ or SiO_2_-P_2_O_5_ oxide glass matrices were investigated and active films prepared; the purpose of the addition of Al or P to the silica matrix was to create non-bridging oxygen species to help disperse the Yb^3+^ ions homogeneously in the glass network, reducing possible concentration quenching phenomena.

## 2. Results

### 2.1. X-Ray Diffraction

**Figure 1:**
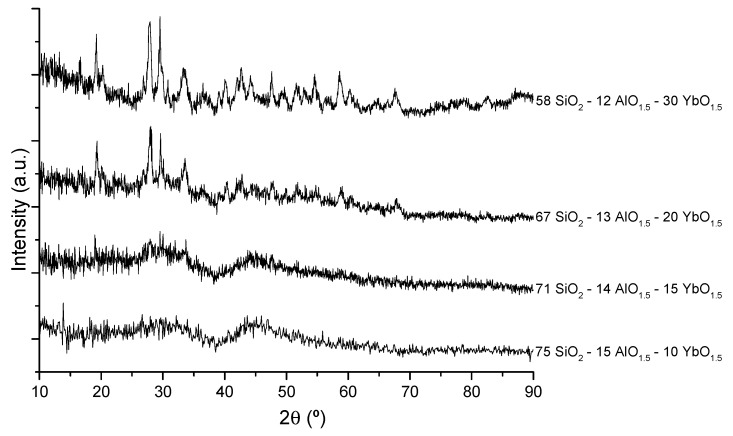
X-ray diffraction patterns of sol-gel derived Yb-doped aluminosilicate bulk samples, heat treated at 1050 °C.

**Figure 2:**
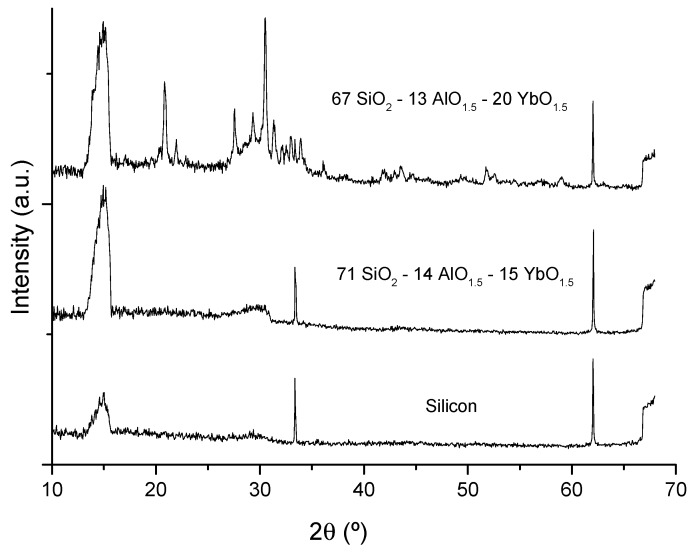
X-ray diffraction patterns of silicon and two aluminosilicate films with 15 and 20 mol% YbO_1.5_, heat treated at 1100 °C.

In order to avoid the presence of scattering centers, it is important to determine whether the heat treatments of the bulk samples (up to ~1050 °C) or the films (up to ~1100 °C) may cause any crystallization. The difference in the temperatures used was due to the fact that multilayer films are treated sequentially, with fast heating from room temperature to the final heat treatment temperature, while bulk samples are monolithic and slowly heated until the final temperature, in order to allow complete combustion of the organic residues. For this purpose, both films and powdered bulk samples were analyzed by XRD. Figure 1 includes the XRD patterns of four aluminosilicate bulk samples, showing the amorphous nature of the aluminosilicates up to 15 mol% YbO_1.5_, whereas higher Yb concentrations led to partial crystallization, namely of the Yb_2_Si_2_O_7_ phase. The same behavior had already been observed for phosphosilicate glass films, where partial crystallization occurred for compositions with more than 5 mol% YbO_1.5_, as reported in [14]. Figure 2 shows the X-ray diffraction (XRD) patterns of Yb-doped aluminosilicate films plus a silicon wafer reference, where partial crystallization is also observed for the 20 mol% YbO_1.5_ film, while the amorphous character of a film with 15 mol% YbO_1.5_ is confirmed. The patterns are truncated at 2θ = 68°, due to the main Si peak at 69.2°, corresponding to the {400} planes.

### 2.2. Raman Spectroscopy

**Figure 3:**
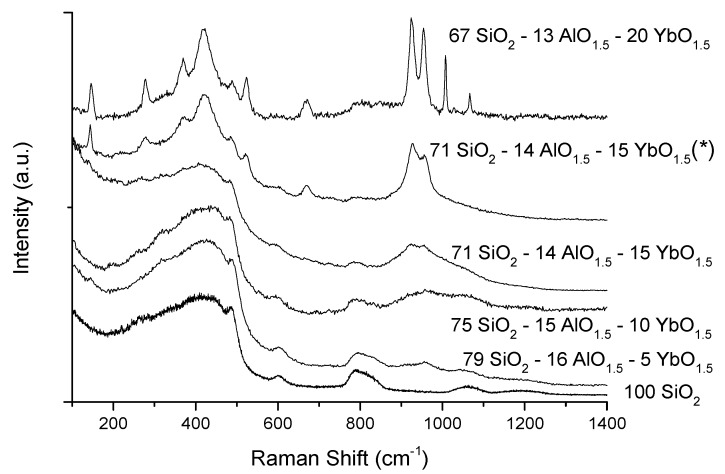
Raman spectra of Yb-doped aluminosilicate bulk glasses. The samples were heat treated at 1050 °C, except for the sample labelled (*) which was heat-treated at 1100 °C.

Figure 3 shows the Raman spectra of the heat treated Yb-doped aluminosilicate bulk glass samples. The Raman spectra, up to 15 mol% ytterbia, are relatively similar to that of pure vitreous silica, with the growth of a peak near 940 cm^−1^ with increasing Yb concentrations, probably due to the formation of Si-O^–^ non-bridging oxygen bonds with the Yb^3+^ ions, rather than Si-O-Al sequences as previously assigned [14], since there is no correlation with the alumina content, which remains practically constant. For heat treated aluminosilicate compositions with higher Yb contents, the appearance of several sharp peaks suggests the formation of one or more crystalline phases, in agreement with the XRD results. However, this behavior depends also on the heat treatment temperature, as observed for the 71 SiO_2_-14 AlO_1.5_-15 YbO_1.5_ samples, where an increase of 50 °C is sufficient to promote significant crystallization for this composition.

### 2.3. Fourier Transform Infrared (FTIR) Spectroscopy

**Figure 4:**
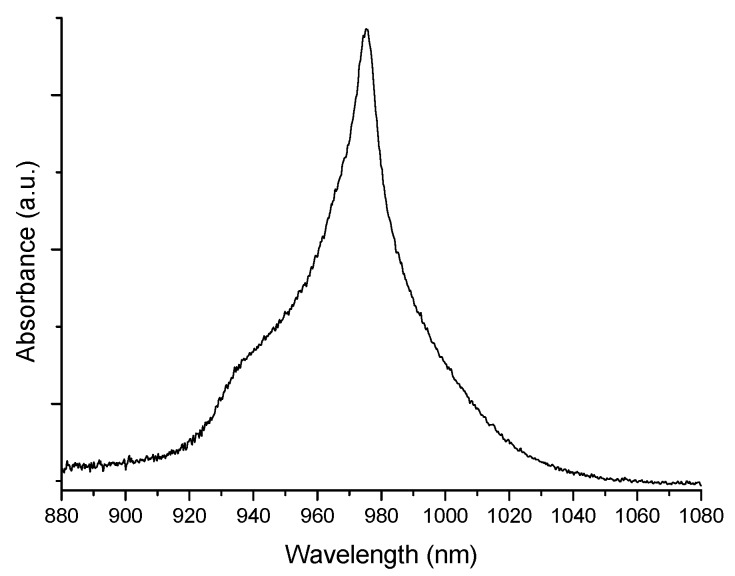
Near infrared absorption spectrum of 79 SiO_2_-16 AlO_1.5_-5 YbO_1.5_ bulk gel.

Yb^3+^ ion absorption was measured in the near infrared (NIR), for both aluminosilicate and phosphosilicate monolithic gels. The Yb^3+^ ion is known to absorb at wavelengths near ~975 nm, due to the ^2^F_7/2_ → ^2^F_5/2_ transition starting from the ground state, with a shoulder near ~940 nm [14]. Figure 4 shows the Yb^3+^ absorption spectrum for the 79 SiO_2_-16 AlO_1.5_-5 YbO_1.5_ bulk gel sample, before heat treatment, when it was still monolithic and transparent.

### 2.4. Photoluminescence Spectroscopy

**Figure 5:**
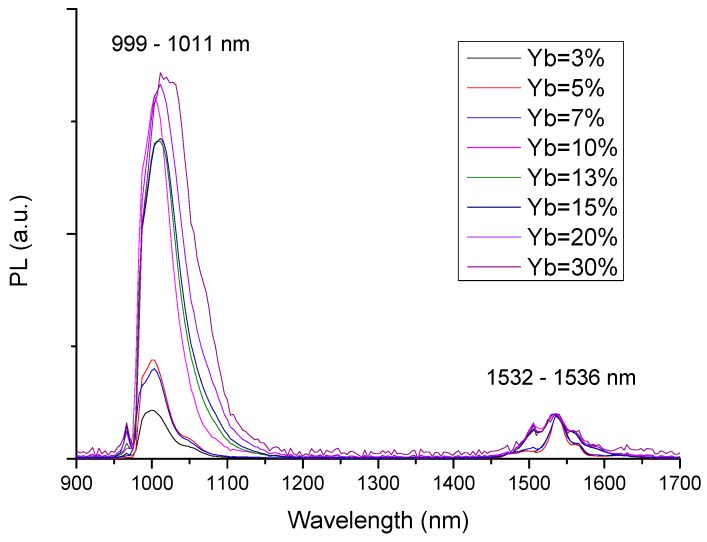
Normalized photoluminescence (PL) emission spectra of Yb/Er co-doped phosphosilicate bulk glass samples, excited at 970 nm.

**Figure 6:**
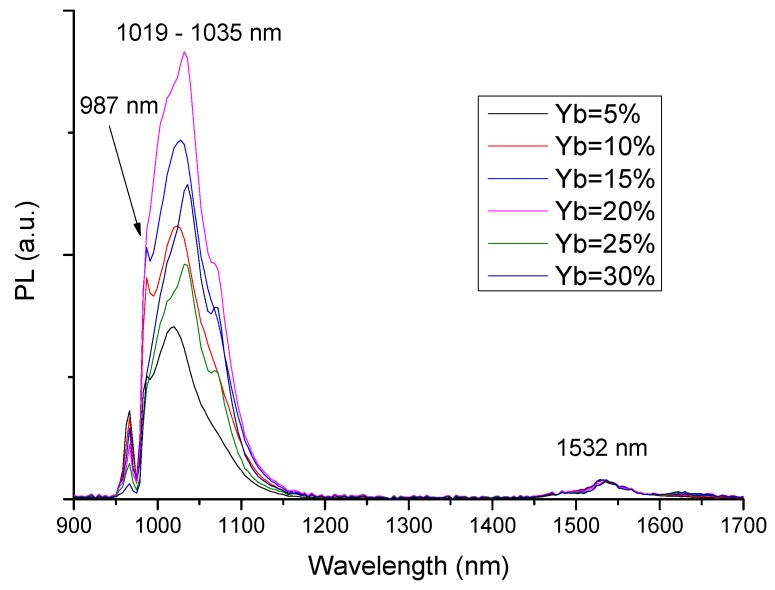
Normalized PL emission spectra of Yb/Er co-doped aluminosilicate bulk glass samples, excited at 970 nm.

Figure 5 and Figure 6 show the photoluminescence (PL) spectra of bulk Yb/Er co-doped phosphosilicate and aluminosilicate-matrix samples, respectively. The spectra have been normalized to the intensity of the Er^3+^ peak emission at 1532 nm. Erbium was used here as an internal reference [13,14] and its concentration, the same in all cases (0.002 mol% Er), was kept to a minimum in order to avoid any significant Yb^3+^ → Er^3+^ energy transfer through the so-called “antenna effect”.

In Figure 5, two main peaks can be observed for the phosphosilicate glasses at 999–1011 and 1532 nm, representing Yb^3+^ and Er^3+^ PL emissions due to ^2^F_5/2_ → ^2^F_7/2_ and ^4^I_13/2_ → ^4^I_15/2_, respectively. The Yb^3+^ PL peak position varied with the Yb content between 999 nm (for 3 mol% Yb) and 1011 nm (for 30 mol% YbO_1.5_). The normalization at 1532 nm revealed a general increase in the PL peak intensity with increasing Yb content. A residual peak is also observed at 970 nm, due to the incompletely filtered excitation laser light.

In Figure 6, the main emission peak of Yb-doped aluminosilicate glasses is located between 1019 and 1035 nm and its intensity increased for Yb contents up to 20 mol%, whereas it decreased for concentrations above that. An additional Yb^3+^ emission peak can be observed at 987 nm, which was not resolved in the phosphosilicate glass spectra of Figure 5.

**Figure 7:**
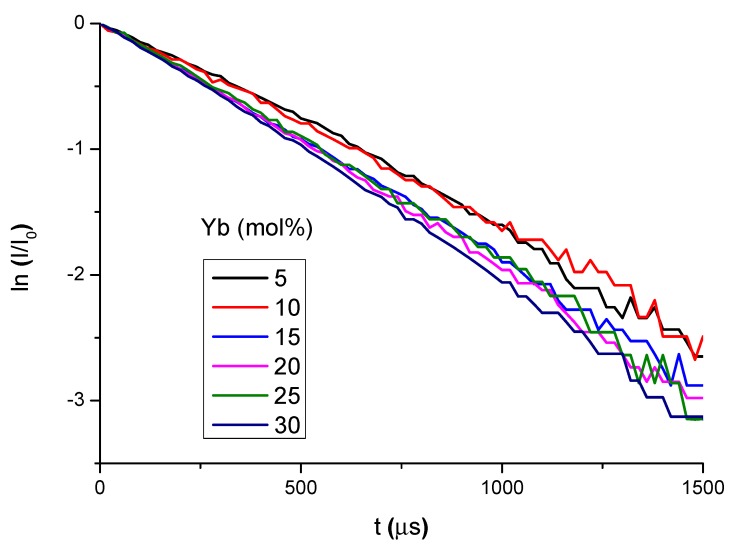
PL decay curves of aluminosilicate bulk glass samples doped with 5–30 mol% YbO_1.5_.

The Yb^3+^:^2^F_5/2_ metastable level lifetimes were also measured for the phosphosilicate and aluminosilicate compositions. The PL decay curves had single exponential behavior as a function of time, t, as shown by the logarithmic plots of Figure 7 for aluminosilicate bulk glass samples doped with 5–30 mol% YbO_1.5_. The corresponding 1/e lifetime, τ, was found to vary between 1043 and 542 μs for phosphosilicates and between 624 and 482 μs for aluminosilicates, as shown in Table 1 and Table 2, respectively.

### 2.5. Active Disk

The active disk may consist of a thin disk (or thick film) of Yb-doped material, sandwiched between a highly reflective coating or Bragg Mirror (BM) and an anti-reflective coating (ARC). The characteristics of the BM, the ARC and active disk were all simulated and designed using the Transfer Matrix Method (TMM) software, developed in our group [11].

The high-reflective coatings, or BMs, were prepared by depositing seven pairs of low/high index layers of aluminosilicate glass (AS) and titania (T), respectively, directly onto the Si substrate. The molar composition 90 SiO_2_-10 AlO_1.5_ was chosen for the low-index AS material, based on previously developed 91 SiO_2_-9 AlO_1.5_ similar compositions [11]. Next, a thick Yb-doped aluminosilicate glass film with the optimized composition (71 SiO_2_-14 AlO_1.5_-15 YbO_1.5_) was deposited, followed by a photonic structure with anti-reflective properties at the top of the active disk. A small number of layers were used for this ARC, in order to avoid damaging the previously deposited Yb-doped multilayer disk through the additional heat treatment routines. The final ARC structure prepared consisted of three layers only (AS/T/AS) deposited on top of the Yb-doped active disk.

#### 2.5.1. TMM Simulations

TMM simulations allowed the calculation of the transmission and reflection of a multilayer structure within a given wavelength range, based on the thickness and refractive index of each individual layer. Such simulations were performed in order to optimize both the BM and ARC, in terms of the number of low/high index pairs and individual layer thickness. The optimized BM consisted of seven pairs of AS (90 SiO_2_-10 AlO_1.5_)/T (TiO_2_) layers. The basic criterion is the “quarter-wave” condition Equation (1) for the optical thickness of each layer (equal to the product of the physical thickness, *x* and the refractive index, *n*) for a peak wavelength *λ*. The thickness of the AS (low index, *n* = 1.46) and T (high index, *n* = 2.22) layers were 175 and 115 nm, respectively, for a BM stop band with a center wavelength *λ* = 1020 nm (near the Yb^3+^ PL emission maximum), according to:

(1) $$\;x=\frac{\lambda }{4n\;}$$

The ARC, on the other hand, designed for optimum anti-reflectivity at a pumping wavelength of 940 nm, was formed by a 42 nm titania layer sandwiched between two 180 nm aluminosilicate glass layers, whose thickness values (not “quarter-wave”) were obtained from TMM simulations by trial and error.

**Figure 8:**
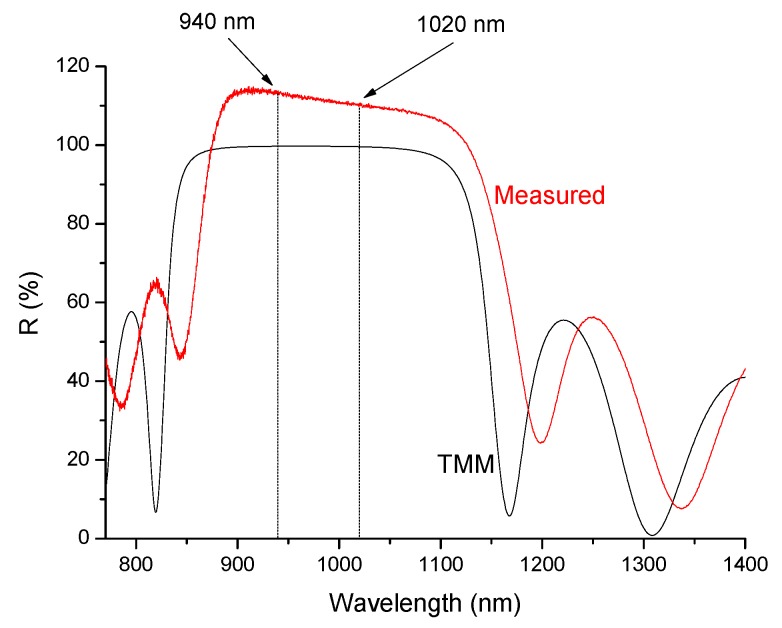
Measured reflectance spectrum of a Bragg Mirror (BM) with 7 pairs of AS/T layers deposited on a Si wafer, compared with Transfer Matrix Method (TMM) simulation.

**Figure 9:**
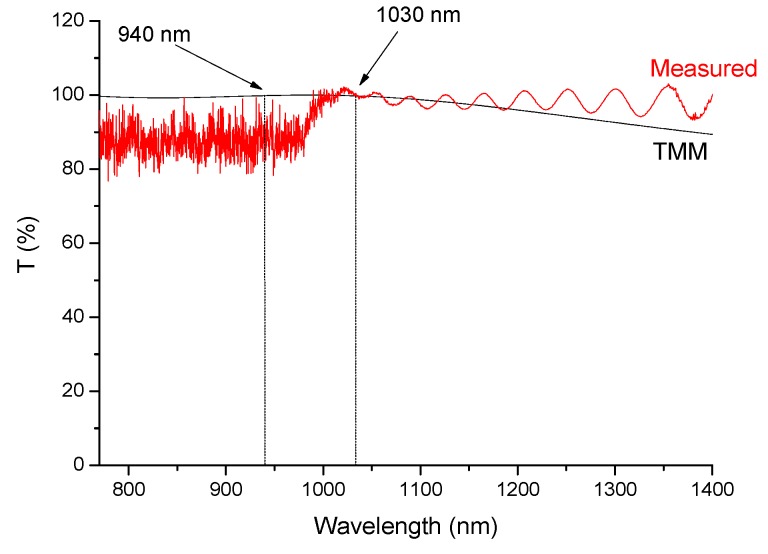
Transmission of an anti-reflective coating (ARC) with 3 layers deposited on 71 SiO_2_-14 AlO_1.5_-15 YbO_1.5_ glass film deposited on silicon substrate, compared with TMM simulation.

Figure 8 compares a BM reflectance measurement with its TMM simulation, while Figure 9 compares the transmission of the ARC with the corresponding TMM simulation. In the former case, high reflectivity is observed at both the 940 nm and the 1020 nm wavelengths; the observed values were slightly higher than 100%, since the BM reflected more than the Al mirror used as reference. In the latter case, the transmission is near 100%, evidencing the ARC properties and the interference fringes are due to the ARC thickness the agreement between theory and experiment appears reasonably good in both cases.

#### 2.5.2. Active Disk Structure

**Figure 10:**
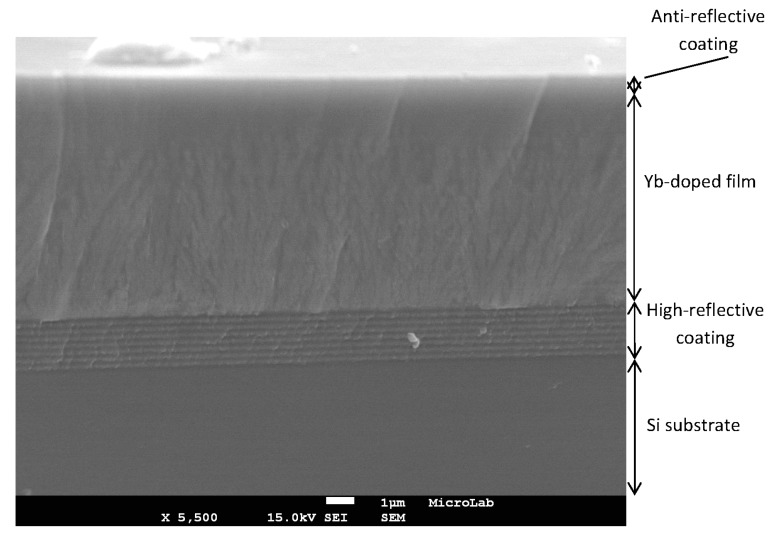
FEG-SEM cross section micrograph of a ~10 μm thick active disk doped with 15 mol% YbO_1.5_, obtained in secondary electron mode. (Scale bar = 1 μm).

An active disk doped with 15 mol% YbO_1.5_, whose structure consisted of a 7-pair BM, an active layer with molar composition 71 SiO_2_-14 AlO_1.5_-15 YbO_1.5_ and a 3-layer ARC, deposited on single-crystal silicon, was observed by scanning electron microscopy (SEM) and Figure 10 shows the corresponding cross-sectional image. This active disk had an overall thickness of ~10 μm. In the highly reflective portion, it is possible to distinguish the individual BM layers, for a total of seven high/low index pairs. In addition, the fracture surface of the Yb-doped portion is typical of an amorphous material, without any visible grains at this magnification.

#### 2.5.3. Active Disk Properties

**Figure 11:**
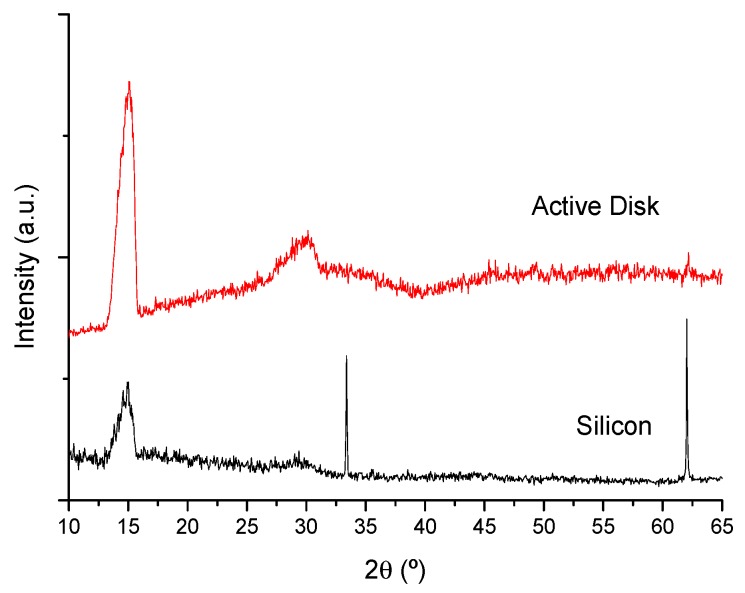
X-ray diffraction patterns of an active disk (BM/36.3 μm thick 71 SiO_2_-14 AlO_1.5_-15 YbO_1.5_ film/ARC) on a silicon substrate.

Figure 11 shows the XRD patterns of a ~36 μm active disk, deposited on silicon, where there is no evidence of any residual crystalline material.

**Figure 12:**
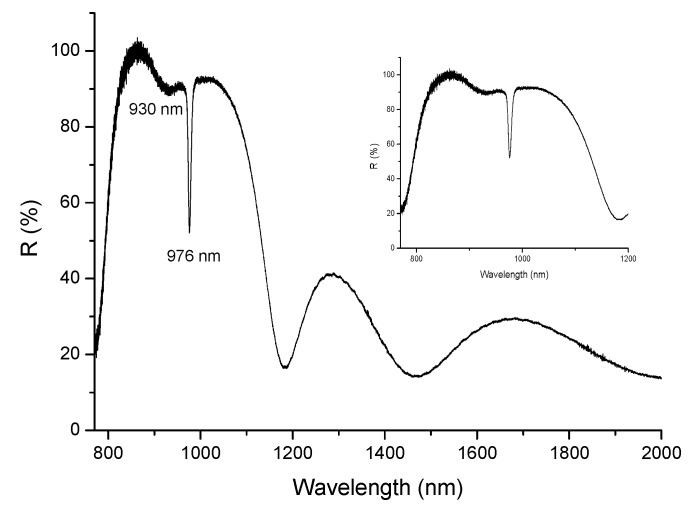
Reflectance of an active disk composed of a BM, an Yb-doped layer (36.3 micron thick) and an ARC coating, deposited on silicon.

The reflectance of the same active disk was measured in the NIR region (Figure 12) and a broad stop band of high reflectivity is visible over the range of ~750–1200 nm, due to the BM. A reflection-absorption effect is also observed at ~900–1000 nm, due to the Yb^3+^ absorption within the active layer.

**Figure 13:**
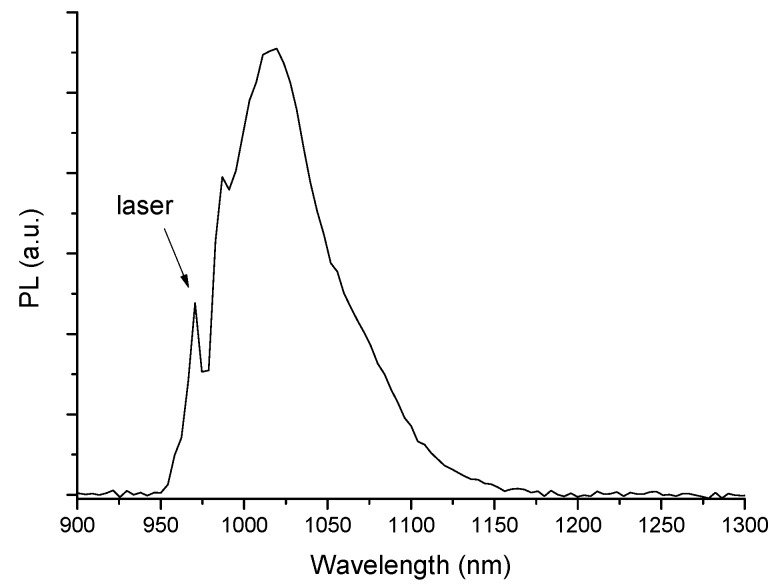
PL emission spectrum of an active disk (BM/71 SiO_2_-14 AlO_1.5_-15 YbO_1.5_ film, 36.3 μm thick/ARC), excited at 970 nm.

The PL spectrum of the active disk is presented in Figure 13, which clearly shows the 987 nm and the major Yb^3+^ PL emission peaks, in addition to the residual excitation laser line.

## 3. Discussion

The SG method used in the present work enables the synthesis of the active material for an Yb-doped thin-disk laser through spin-coating deposition of disk-like films more than 30 μm thick. While the nature of the Yb-doped material was assessed by XRD plus FTIR and Raman spectroscopies, the morphology of the multilayer films and their optical quality were determined based on SEM analysis. The PL emission spectra and Yb^3+^ metastable level lifetimes of the doped material were also studied in detail.

XRD and Raman data indicate the amorphous nature of these aluminosilicate bulk samples and films with up to 15 mol% Yb, reveal a partially crystalline nature for 20 to 30 mol% Yb. However, an increase of the final heat treatment temperature of the bulk glasses from 1050 to 1100 °C is enough to promote some crystallization in the composition with 15 mol% Yb. Therefore, the Yb content, the glass matrix, and the heat treatment conditions are critical for the amorphous character of the samples. In fact, while aluminosilicate compositions remain amorphous for up to 15 mol% Yb, the phosphosilicates become partially crystallized with just 5 mol% Yb [14], revealing the different capabilities of Al and P to disperse the Yb^3+^ ions in the matrix, since the pentavalent P is less easily incorporated in the silicate glass structure compared to Al, which readily substitutes for Si.

The PL emission peaks at 987 and 1019–1035 nm correspond to transitions between the Stark levels of the ^2^F_5/2_ excited state and the ^2^F_7/2_ ground state of Yb^3+^: the sharp peak at 987 nm corresponds to the transition 5 → 1, between the lowest Stark levels of each J-manifold, whereas the intense peak at 1019–1035 nm is attributed to longer wavelength transitions from level 5 to higher lying Stark levels of the ground state, followed by thermalization to the lower J-level [15], without well resolved Stark splitting due to the amorphous nature of the host matrix. The continuous decrease of the measured PL lifetime with increasing Yb content, especially in the case of the phosphosilicate glass matrix (Table 1), indicates the occurrence of concentration quenching phenomena. This effect was already observed in previous studies [14,16,17], involving non-radiative energy transfer phenomena between closely spaced Yb^3+^ ions, despite the common assumption that the simple electronic energy level structure of Yb^3+^ excludes Excited State Absorption and a variety of quenching processes [18]. While the PL spectra (Figure 5 and Figure 6) refer to Yb/Er co-doped samples, the lifetime values were measured in Yb-doped samples without Er, to avoid any possible influence of Yb^3+^ → Er^3+^ energy transfer phenomena on the measured lifetimes. It is important to add that most samples were in the form of small plates, a few millimeters on the sides and a fraction of a mm thick. Since this area was still much larger than the laser spot size (typically of the order of ~0.5 mm), we did not observe any size effects. We also did not consider the possibility of self-absorption and photon trapping phenomena of the type reported by Mattarelli et al. [19] and Koughia and Kasap [20], given the fact that the samples were opaque and quite thin.

The potential performance of the present Yb-doped sol-gel thick films as thin-disk laser materials was evaluated based on the PL results, through a figure of merit (FOM) defined as the στ product between the normalized spontaneous emission cross section σ, taken as the ratio of Yb^3+^ (1000–1035 nm)/Er^3+^ (1532 nm) PL peak intensities and the corresponding ^2^F_5/2_ metastable level lifetime, τ. Table 1 and Table 2 indicate that the aluminosilicate composition 67 SiO_2_-13 AlO_1.5_-20 YbO_1.5_ had the highest FOM. However, XRD data (Figure 1 and Figure 2) show the occurrence of some incipient crystallization in this material. Therefore, the aluminosilicate composition 71 SiO_2_-14 AlO_1.5_-15 YbO_1.5_, which is completely amorphous and had the second highest FOM value, is chosen as the best composition for the doped film portion of the active disk. It is expected that an FOM based on stimulated emission cross-sections would lead to a similar conclusion.

## 4. Materials and Methods

### 4.1. Sample Preparation

Heavily Yb-doped aluminosilicate and phosphosilicate glass films and monoliths were prepared by the acid (HCl) catalyzed SG method, with the compositions shown in Table 3, where the aluminosilicate and phosphosilicate matrices had Si:Al and Si:P molar ratios approximately constant and equal to ~5:1 in both cases in order to keep a similar starting glass matrix structure, while the YbO_1.5_ content varied between 3 and 30 mol% on a cation basis. A small quantity of Er (0.002 mol%) was added as an internal reference for the PL measurements [13,14]. The precursors used for silica, phosphorus oxide, alumina, ytterbia, and erbia were tetraethyl orthosilicate (Alfa Aesar, Karlsruhe, Germany, 98%), P_2_O_5_ (Sigma-Aldrich Gmbh, Munich, Germany, ≥98%), aluminium nitrate nonahydrate (Alfa Aesar, Karlsruhe, Germany, 98%–102%), ytterbium nitrate pentahydrate (Strem Chemicals, Bischheim, France, 99.9%) and erbium nitrate pentahydrate (Aldrich Chemical Co., Inc., Milwaukee, WI, USA, 99.9%), respectively. Processing details for these materials are given elsewhere [14]. Titania films, used as high index material in the photonic bandgap (PBG) structures, were also synthetized by the SG method, using titanium (IV) isopropoxide (TiPOT) (Alfa Aesar, Karlsruhe, Germany, ≥97%) as precursor. Acid catalysis was also used here, through the mixing of glacial acetic acid (GAA), (Merck, Darmstadt, Germany, 100%) with TiPOT and stirring at room temperature over 1 h. After mixing, ethanol (Merck, ≥99.5%) was added slowly and the titania solution was stirred at room temperature for an additional hour.

#### 4.1.1. Bulk Sample Preparation

Transparent silicate glass monoliths were obtained by aging the final SG solution for several days in an open container, followed by a heat-treatment of the obtained gel up to 1050 °C at a rate of 0.5 °C/min and slow cooling inside the furnace. During the heat treatment, most samples crumbled into a powder.

#### 4.1.2. Film Deposition

Silicate or titania precursor solutions were aged for at least 24 h and 3 h, respectively, in a closed container and they were then spin-coated onto single crystal Si wafer substrates, at 2000 rpm for 30 s, using a Chemat KW-4A spin-coater. For thick multilayer deposition, each individual layer was heat treated at 1100 °C for 30 s in a muffle furnace.

#### 4.1.3. Active Disk Preparation

**Figure 14:**
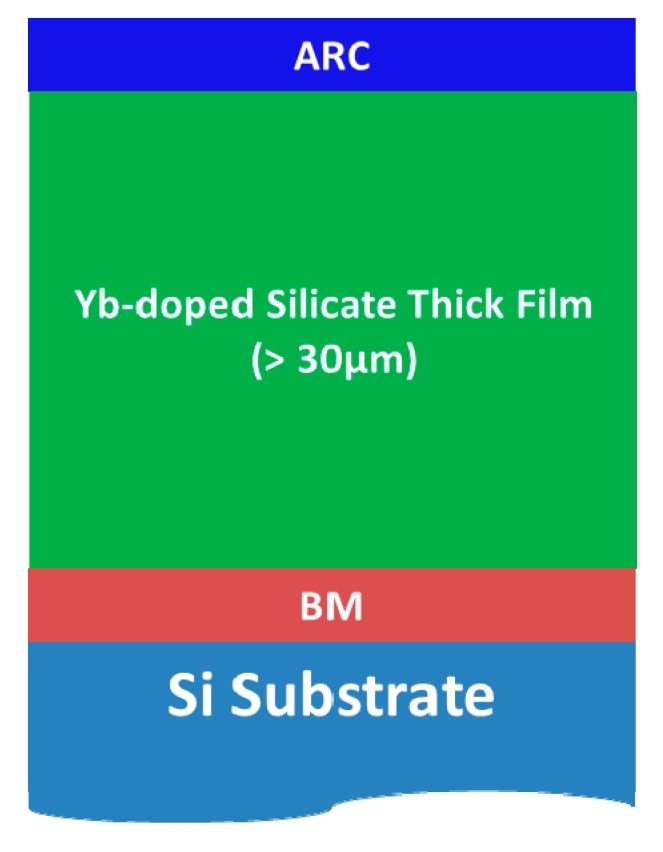
Schematic structure of the active disks prepared.

In a Yb thin-disk laser, the active element may consist of a thin disk (or thick film) of Yb-doped material, sandwiched between a highly reflective coating or Bragg Mirror with peak reflectance at the signal wavelength (~1020 nm) and an anti-reflective coating with lowest reflectance at the pump wavelength (usually ~940 nm). When prepared by SG, the active disk will be a multilayered structure formed by several individual layers deposited in sequence according to the scheme shown in Figure 14.

### 4.2. Sample Characterization

Bulk powdered samples and thick films were analyzed by X-ray diffraction (XRD) with a Philips PW3020 powder diffractometer, at room temperature, using Cu Kα radiation (λ = 1.541874 Å) generated at 40 kV and 30 mA, in the 2θ range of 10°–90°, with a step of 3°/min. The average crystal size was estimated by X-ray line broadening, using the Scherrer formula [21] and taking into account the instrumental broadening.

Surface and cross-section images of active disk samples were obtained by Scanning Electron Microscopy (SEM) using a 7001F FEG-SEM (JEOL, Zaventem, Belgium) in secondary and/or back-scattered electron modes, at 15 kV. A ~15 nm layer of chromium was deposited onto the film samples, in order to promote the electrical conductivity at the surface of the insulating materials under observation.

A Nicolet 5700 FT-IR spectrometer (Thermo Electron Corporation, Madison, WI, USA) was used to record near infrared spectra, at a resolution of 4 cm^−1^. The 975 nm Yb^3+^ absorption peak was recorded in the NIR, in transmission, for transparent glass monolithic samples and in reflection mode, for highly reflective and ARC layers deposited on silicon wafers.

Raman spectra were collected with a LabRam HR 800 Evolution confocal micro-Raman spectrometer (Horiba, Villeneuve d’Ascq, France). The measurements were carried out with 532 nm laser excitation, using a 600 gr/mm grating and 100× objective lens. The incident laser power on the samples was ~10 mW and the spot diameter was ~1 µm.

The PL of Yb-doped silicate materials was excited with a 970 nm laser (Lumics High Power Module), using 3 W power and a filter to block the laser radiation. The emitted light was analyzed by an Avaspec-NIR256-1.7 fiber-optic spectrometer (Avantes, Apeldoorn, The Netherlands), at a resolution of 4 nm. The Yb^3+^ PL lifetimes were measured for chopped light with a PM3370B 60 MHz digital oscilloscope (Fluke, Eindhoven, The Netherlands).

## 5. Conclusions

A Ytterbium-doped active material for a thin-disk laser was developed using sol-gel processing. Ytterbium doped aluminosilicate and phosphosilicate glass matrices were investigated with a doping level between 3 and 30 mol% YbO_1.5_. Films with a thickness up to ~36 μm were prepared by spin-coating and bulk samples were obtained by sol-gel processing as well. The amorphous nature of the materials prepared was assessed by XRD and Raman spectroscopy, with some incipient crystallization observed in aluminosilicate samples with ≥20 mol% Yb and in phosphosilicate samples with ≥5 mol% Yb. The PL properties of Yb^3+^ were measured, with lifetime values between 1.0 and 0.5 ms when the Yb concentration increased from 3 to 30 mol%, with a concentration quenching effect for >3 mol% Yb. The Yb^3+^ PL intensities at λ ~1 μm, normalized to that of Er^3+^ internal standard at ~1.5 μm, together with the lifetime values, were combined in an FOM which led to the selection of the 71 SiO_2_-14 AlO_1.5_-15 YbO_1.5_ composition (mol%) as the best material for the active layer. An active disk ~36 μm thick, with a structure consisting of a Bragg mirror, an aluminosilicate layer doped with 15 mol% Yb and an anti-reflective coating was fabricated.

## Figures and Tables

**Table 1:** Lifetime, τ and σ × τ values for Yb-doped phosphosilicate glasses.

YbO_1.5_ (mol%)	τ (μs)	σ × τ (μs)
3	1043	1126
5	782	1718
7	763	1528
10	735	5931
13	828	5845
15	586	4173
20	485	4039
30	542	4652

**Table 2:** Lifetime, τ and σ × τ values for Yb-doped aluminosilicate glasses.

YbO_1.5_ (mol%)	τ (μs)	σ × τ (μs)
5	624	5509
10	614	8586
15	525	9648
20	511	11699
25	528	6349
30	482	7762

**Table 3:** Rare-earth doped phosphosilicate and aluminosilicate glass compositions (mol%).

SiO_2_	PO_2.5_	AlO_1.5_	YbO_1.5_
81	16	−	3
79	16	−	5
77	16	−	7
75	15	−	10
73	14	−	13
71	14	−	15
67	13	−	20
58	12	−	30
79	−	16	5
75	−	15	10
71	−	14	15
67	−	13	20
63	−	12	25
58	−	12	30
